# Supplementary material for: Optimal dimensionality selection for independent component analysis of transcriptomic data
Source: BMC Bioinformatics. 2021 Dec 8;22:584. doi: 10.1186/s12859-021-04497-7 (PMC8653613; doi:10.1186/s12859-021-04497-7)
Supplement: Supplementary file 1 — Additional file 1: Figure S1. Due to density-based clustering of ICA run with randomized restarts, the number of robust components do not directly correlate with the selected dimensionality. Figure S2. Across all dimensions components revealed in the final decomposition were found to be well conserved at the established threshold. All components in the final decomposition were correlated pairwise with those of all preceding decompositions. Once a component present in the final decomposition was calculated at a lower dimension that component continued to persist within decompositions of higher dimensionality. A component in the final decomposition was said to be present in a particular decomposition where its correlation with a component in that decomposition was above the established threshold. Across all datasets, PRECISE 1.0, PRECISE 2.0, B. subtilis, and StaphPRECISE, components were found to be well conserved, rarely dropping below their established threshold once computed at a lower dimension. PRECISE 1.0 shown here for example. Figure S3. Components from the final, fully decomposed dataset were correlated pairwise with components of all preceding decompositions. Histograms of the highest correlations for each component across all dimensions were plotted for (A) PRECISE 1.0, (B) PRECISE 2.0, (C) B. subtilis, and (D) StaphPRECISE. The elbow point of these highly correlated values served as the threshold to classify a particular component as conserved. [file 12859_2021_4497_MOESM1_ESM.docx]

# Supplementary Figures


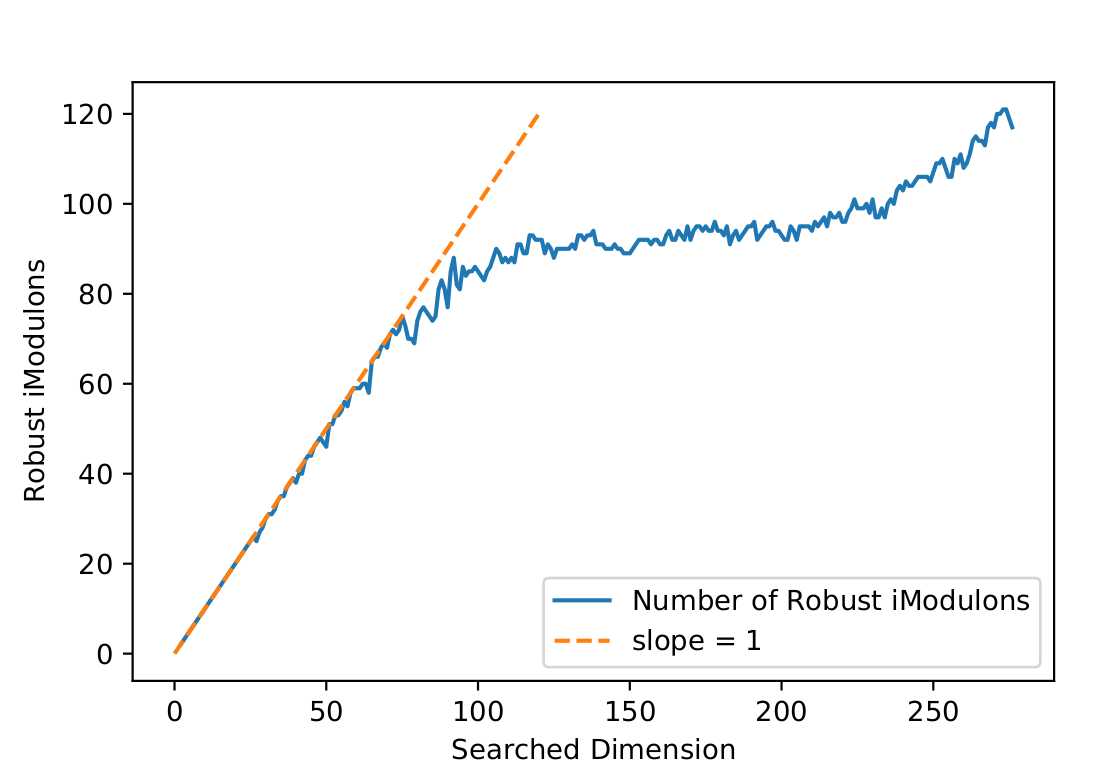


**Supplementary Figure 1.** Due to density-based clustering of ICA run with randomized restarts, the number of robust components do not directly correlate with the selected dimensionality.


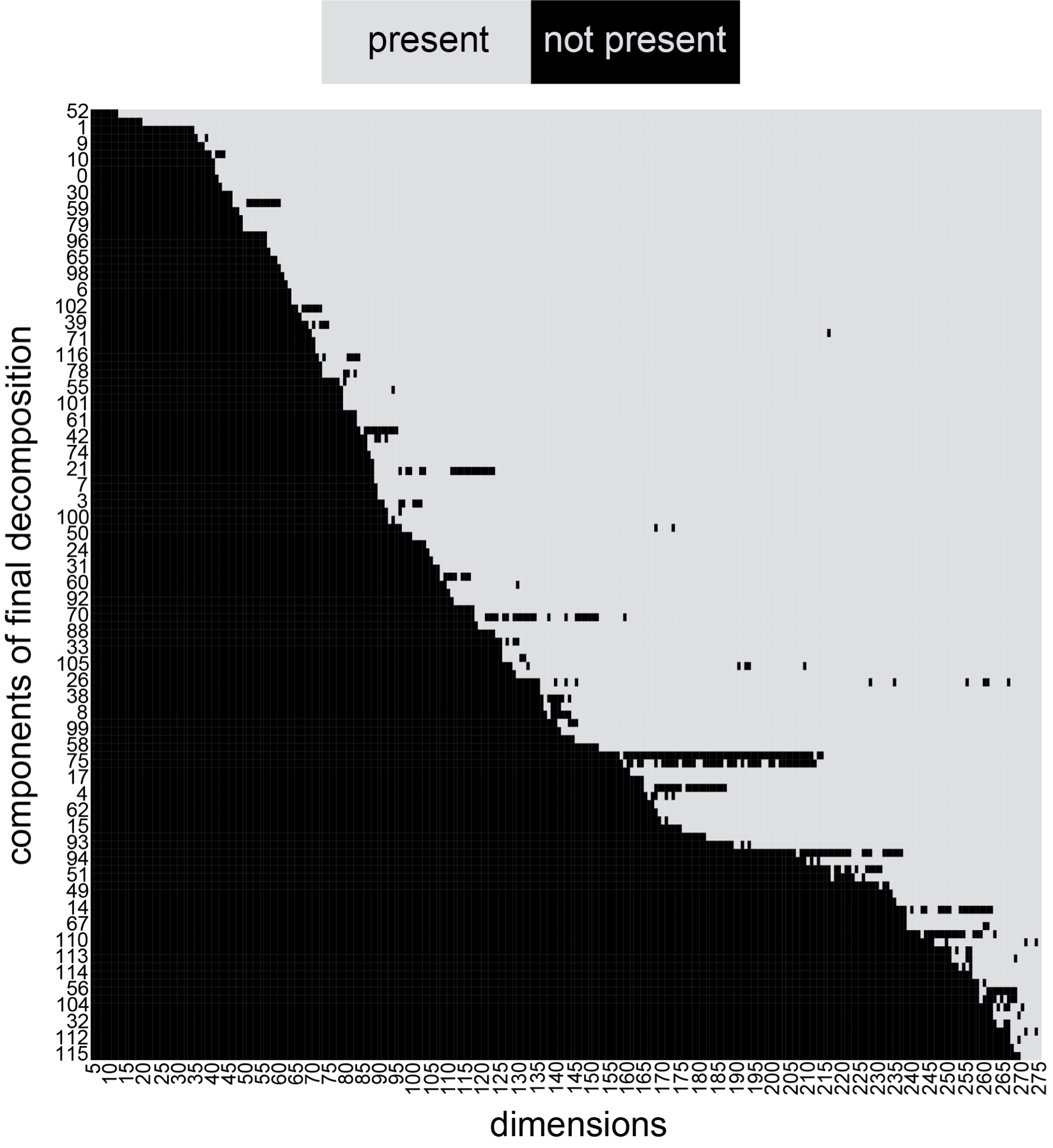


**Supplementary Figure 2.** Across all dimensions components revealed in the final decomposition were found to be well conserved at the established threshold. All components in the final decomposition were correlated pairwise with those of all preceding decompositions. Once a component present in the final decomposition was calculated at a lower dimension that component continued to persist within decompositions of higher dimensionality. A component in the final decomposition was said to be present in a particular decomposition where its correlation with a component in that decomposition was above the established threshold. Across all datasets, PRECISE 1.0, PRECISE 2.0, *B. subtilis*, and *Staph*PRECISE, components were found to be well conserved, rarely dropping below their established threshold once computed at a lower dimension. PRECISE 1.0 shown here for example.


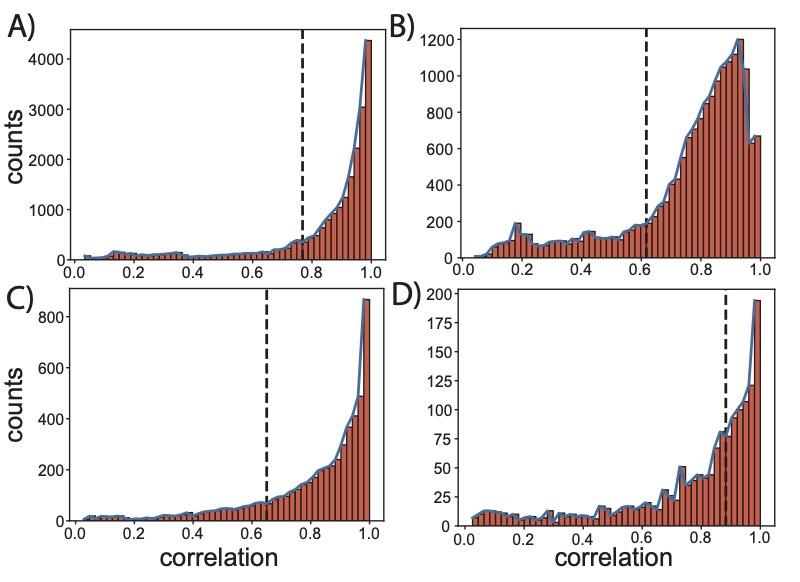


**Supplementary Figure 3.** Components from the final, fully decomposed dataset were correlated pairwise with components of all preceding decompositions. Histograms of the highest correlations for each component across all dimensions were plotted for (A) PRECISE 1.0, (B) PRECISE 2.0, (C) *B. subtilis*, and (D) *Staph*PRECISE. The elbow point of these highly correlated values served as the threshold to classify a particular component as conserved.
